# Poor neural and perceptual phoneme discrimination during acoustic variation in dyslexia

**DOI:** 10.1038/s41598-020-65490-3

**Published:** 2020-05-26

**Authors:** P. Virtala, S. Talola, E. Partanen, T. Kujala

**Affiliations:** 1 Cognitive Brain Research Unit, Department of Psychology and Logopedics, Faculty of Medicine, University of Helsinki, Helsinki, Finland; 2 Cognitive Brain Research Unit, Institute for Behavioural Sciences, University of Helsinki, Helsinki, Finland; 3 Center of Functionally Integrative Neuroscience (CFIN), Department of Clinical Medicine, Aarhus University, Aarhus, Denmark

**Keywords:** Cortex, Dyslexia, Language

## Abstract

Whereas natural acoustic variation in speech does not compromise phoneme discrimination in healthy adults, it was hypothesized to be a challenge for developmental dyslexics. We investigated dyslexics’ neural and perceptual discrimination of native language phonemes during acoustic variation. Dyslexics and non-dyslexics heard /æ/ and /i/ phonemes in a context with *f*_o_ variation and then in a context without it. Mismatch negativity (MMN) and P3a responses to phoneme changes were recorded with electroencephalogram to compare groups during ignore and attentive listening. Perceptual phoneme discrimination in the variable context was evaluated with hit-ratios and reaction times. MMN/N2bs were diminished in dyslexics in the variable context. Hit-ratios were smaller in dyslexics than controls. MMNs did not differ between groups in the context without variation. These results suggest that even distinctive vowels are challenging to discriminate for dyslexics when the context resembles natural variability of speech. This most likely reflects poor categorical perception of phonemes in dyslexics. Difficulties to detect linguistically relevant invariant information during acoustic variation in speech may contribute to dyslexics’ deficits in forming native language phoneme representations during infancy. Future studies should acknowledge that simple experimental paradigms with repetitive stimuli can be insensitive to dyslexics’ speech processing deficits.

## Introduction

Adequate language skills are essential for human communication, but also for cognitive development and academic achievement. Language development starts when the infant is exposed to native language speech and learns to extract meaningful units like phonemes and words from it. This learning is challenging as the acoustical features of phonemes differ between individual speakers, and within each speaker due to, for example, prosodic variation and surrounding phonemes. In order to learn and efficiently perceive the phoneme categories of one’s native language^1^, the human brain must tolerate this variability while still being sensitive to differences between phonemes. Studies on event-related potentials Event-related potentials (ERPs) recorded with magnetoencephalography (MEG) and electroencephalography (EEG) have suggested that not only adults^2^ but even newborn infants^3^ might have this ability. When presented with a sound stream of different native language phonemes uttered by several speakers, mismatch negativity (MMN) was found in adults and newborns to phoneme changes (its magnetic counterpart MMNm^2^ and its infant counterpart mismatch response, MMR^3^). This was interpreted as evidence of neural extraction of phoneme categories^2,3^.

Although the brain of typically developing individuals automatically and effortlessly discriminates changes in phonemes even in a variable context, this ability can be compromised in various developmental disorders. The most prevalent, heritable language-related developmental disorder is developmental dyslexia that hampers the acquisition of a fluent reading skill^4,5^, affecting up to 5–17% of children^6,7^. Dyslexia stems from various neurodevelopmental structural and functional abnormalities^8–10^ and is currently thought to be mainly based on a deficit in forming robust phonological representations during native-language acquisition^8,11,12^. Alternatively or additionally, dyslexia might result from poor access to these representations^11,13^.

The phonological processing deficit hypothesis in dyslexia has gained support from studies using the MMN as an index of speech-sound representations and sound-discrimination accuracy^14–16^. The MMN is an ERP component with a negative polarity elicited 100–250 ms after stimulus change onset in a repetitive sound stream reflecting pre-attentive sound discrimination^17,18^. When the deviance is salient enough to attract the listener’s attention, the MMN is typically followed by a negative N2b, these two responses forming an “N2” response^19^, and a positive P3a component^20,21^. The MMN is enhanced to native versus foreign language phonemes, reflecting memory traces of the native-language speech sounds, i.e., phoneme representations^22,23^. While robust MMNs are associated with well-formed phonological representations and language skills, weak, absent, delayed, or atypically lateralized MMN responses are connected with language disorders^16,24^. Furthermore, these abnormal MMNs may predict future language and reading abilities^24–26^, suggesting that they have a strong association with language-related skills.

In addition to diminished MMN amplitudes to changes in speech sounds^27–31^, dyslexics have problems in later, attentive, stages of speech processing. For example, whereas dyslexics were found to discriminate large duration changes in pseudowords in an intact manner as evidenced by normal-like MMN amplitudes, they failed to identify their position in an attentive detection task, as reflected by an absent N2b response^32^. Furthermore, unlike control participants, dyslexics failed to demonstrate a P300/P3a enhancement to phoneme changes in words and pseudowords, interpreted to reflect attentional deficits in phoneme detection in dyslexia^33^.

Dyslexia has also been associated with broader auditory difficulties, namely in processing acoustic changes in non-speech sounds^34,35^. These studies have suggested that small stimulus differences are particularly challenging for the auditory system of dyslexic individuals^14,35,36^. Furthermore, temporal or order changes in sound pairs elicited MMNs with normal-like amplitudes in dyslexic participants, whereas adding sounds around the pairs diminished the MMN more in dyslexic than control participants^37,38^. This was interpreted as a sensory memory deficit involving elevated masking effects of the surrounding sounds in dyslexics^38^. The result can also be considered as compromised auditory processing in dyslexia when the context is complex and variable. Accordingly, in a so-called multi-feature paradigm with several auditory deviants presented in the same experiment, dyslexics demonstrated smaller MMNs than in a simple, repetitive oddball paradigm^39^. The authors concluded that the acoustic variation in a multi-feature paradigm compromises dyslexics’ processing and can thus provide a sensitive measure for the auditory deficits in dyslexia.

Even though paradigms with a variable sound context, resembling natural speech, may thus be more sensitive to tap into the auditory deficits in dyslexia, most neurophysiological studies have so far investigated the neural basis of dyslexia with repetitive stimuli. MMN studies considered to tap phoneme discrimination have typically included single exemplars of two phonemes, one serving as the repetitive standard and the other one as the rare deviant stimulus (e.g., /da/ vs. /ga/^31^). In the light of results presented above, such stimulation might not be sensitive for dysfunctions of the auditory system in dyslexia. Furthermore, dyslexics have problems in the categorical perception of phonemes in identification or discrimination tasks in which two phonemes are presented along a continuum from one to the other^40–43^. This categorical phoneme perception deficit might not be captured by MMN studies as described above, where the processing of basic acoustic features such as the pitch of the second formant could be sufficient for discriminating between the two repetitive speech sounds. In contrast, in natural, acoustically variable speech, auditory features have to be grouped together in order to discriminate between phonemes, thus calling for categorical perception.

The aim of the present study was to determine neural and perceptual phoneme processing in dyslexia in a speech sound context with continuous variation. We used very distinct vowels, Finnish /i/ *vs*. /æ/, presented in pairs /i/-/i/ or /i/-/æ/ with acoustic variation accomplished by presenting the pairs from several predefined *f*_o_ levels (variable context) mimicking natural speech (e.g., speaker differences). The paradigm also included another change type, which was not included in the analyses of the present study. MMN and P3a were recorded to the phoneme changes (from /i/ to /æ/) from dyslexics and non-dyslexic controls during ignore and attentive listening conditions (VariableIgnore and VariableAttend, respectively). During attentive listening, the MMN was expected to be followed and/or overlapped by N2b, and therefore two different latency windows were used to try to differentiate between the two responses: one for MMN, and one for MMN/N2b. Participants were also queried about their explicit awareness of the nature of the stimuli in the variable context, both after the VariableIgnore condition, and after VariableAttend condition during a short familiarization sequence (VariableFamiliarization). Perceptual phoneme discrimination was studied separately in the variable context, quantified as hit-ratios and reaction times to phoneme changes. At the end of the experiment, we also presented the vowels without fo-variation (constant context) in an ignore condition (labeled: ConstantIgnore). The specific research questions were: (1) is neural and perceptual phoneme discrimination compromised in a variable auditory context in dyslexia, (2) do dyslexics have difficulties both in attentive and pre-attentive listening conditions, and (3) is neural phoneme discrimination compromised in a constant auditory context in dyslexia.

While dyslexics have problems in discriminating small sound differences in simple contexts, it can be predicted based on earlier findings^39^ that even quite salient sound differences might be challenging for them in a more variable context resembling natural speech. Accordingly, we expected no or small group differences in the neural discrimination of the large phoneme difference in ConstantIgnore condition. However, when presented in an acoustically variable context, we predicted poorer phoneme discrimination in dyslexic than typically reading participants, evident as smaller MMN/N2b amplitudes in both VariableIgnore and VariableAttend conditions as well as lower hit-ratios and longer reaction times. Since an earlier study showed attentional deficits in phoneme processing in dyslexics^33^, we also expected a diminished P3a in dyslexic participants in ignore and attentive listening conditions.

## Results

### Neural phoneme discrimination

**Figure 1:**
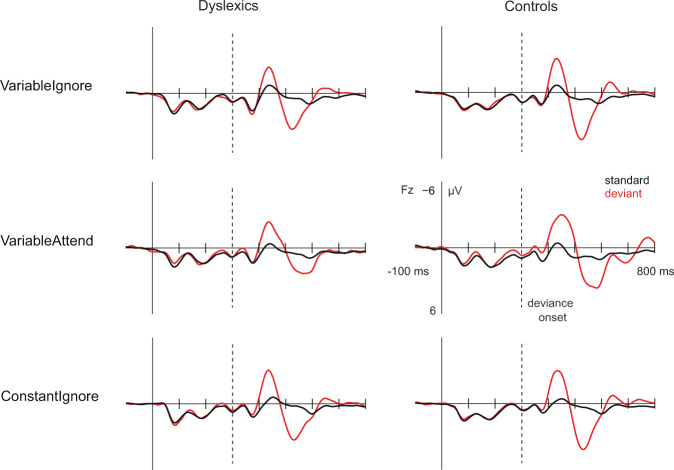
ERPs to standard (black line) and deviant (red line) stimuli in VariableIgnore (top panel), VariableAttend (mid panel) and in ConstantIgnore (bottom panel) at Fz electrode. Dashed vertical line depicts the deviance onset (300 ms). Ten control participants’ data from the variable paradigm were reported in^44^.

**Figure 2:**
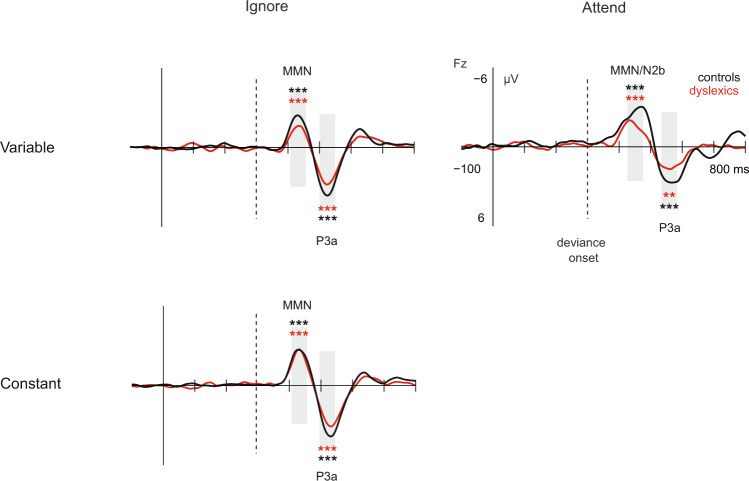
Subtraction curves depicting MMN, MMN/N2b and P3a responses in VariableIgnore, VariableAttend, and ConstantIgnore at Fz electrode. Grey bars mark the time windows for mean amplitudes. Amplitudes differing statistically significantly from 0 in one-sample t-tests are marked with asterisks: ***p < 0.001, **p < 0.01. The dashed vertical line denotes deviance onset (300 ms). Ten control participants’ data from the variable paradigm were reported in^44^.

**Figure 3:**
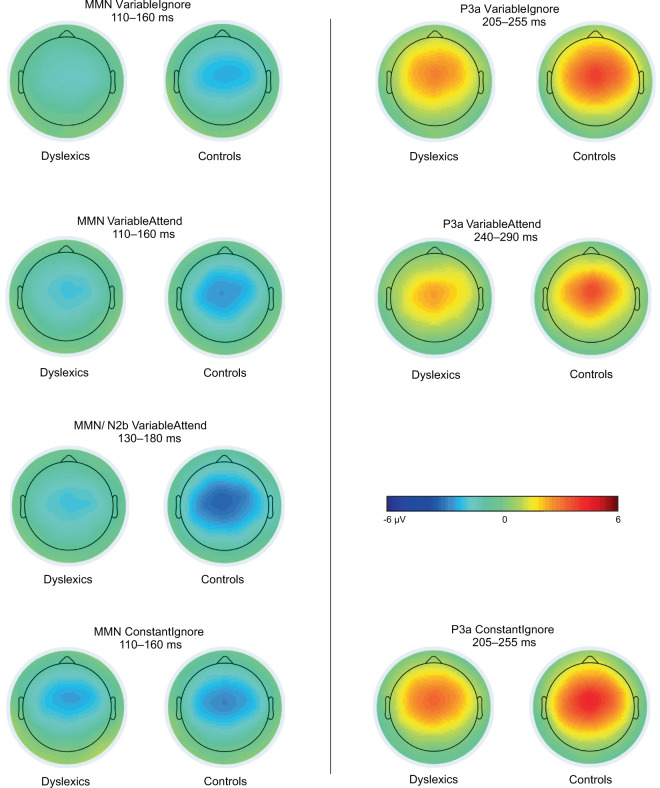
Voltage maps showing scalp distributions of MMN, MMN/N2b, and P3a mean amplitudes on the defined latency windows in dyslexics and controls in the three sequences. Ten control participants’ data from the variable paradigm were reported in^44^.

The ERP waveforms to standard /i/-/i/ and deviant /i/-/æ/ phoneme pairs are shown in Fig. 1, subtraction curves illustrating the MMN/N2b and P3a responses in Fig. 2, and scalp distributions of MMN/N2b and P3a responses in Fig. 3. In VariableIgnore, phoneme changes elicited a statistically significant MMN and P3a in both groups (one-sample t-tests reported in Table 1). According to group effects in repeated-measures analyses of variance (RM-ANOVAs), MMN amplitude was statistically significantly smaller in dyslexics than controls, F(1,36) = 4.98, p = 0.03, ηp^2^ = 0.12, and P3a amplitude tended to be smaller in dyslexics than controls, F(1,36) = 2.97, p = 0.09, ηp^2^ = 0.08.

**Table 1:** The MMN, MMN/N2b and P3a time windows (ms), peak latencies (ms), and mean amplitudes (μV) in VariableIgnore, VariableAttend, and ConstantIgnore on Fz electrode.

Condition	Response	Time window	Group	Peak latency	Mean amplitude
VariableIgnore	MMN	110–160	Dyslexics	130.56 (13.70)	−1.35 (0.87)***
			Controls	135.16 (16.20)	−2.06 (1.09)***
	P3a	205–255	Dyslexics	230.60 (25.64)	2.45 (1.45)***
			Controls	227.15 (19.45)	3.18 (1.44)***
VariableAttend	MMN	110–160	Dyslexics		−1.74 (1.02)***
			Controls		−2.29 (1.11)***
	MMN/N2b	130–180	Dyslexics	152.04 (33.65)	−1.62 (1.08)***
			Controls	152.73 (30.78)	−2.72 (1.40)***
	P3a	240–290	Dyslexics	263.58 (37.89)	1.53 (1.52)**
			Controls	258.20 (34.45)	2.60 (2.17)***
ConstantIgnore	MMN	110–160	Dyslexics	129.92 (10.74)	−2.22 (1.09)***
			Controls	139.70 (35.03)	−2.39 (1.33)***
	P3a	205–255	Dyslexics	235.61 (17.46)	2.72 (1.32)***
			Controls	228.08 (12.37)	3.41 (1.83)***

In VariableAttend, phoneme changes elicited a statistically significant MMN and an MMN/N2b in both groups (Table 1). According to a group effect in the RM-ANOVA, MMN/N2b amplitude was statistically significantly smaller in dyslexics than controls, F(1,36) = 6.67, p = 0.01, ηp^2^ = 0.16. No significant group differences were found for the MMN (p = 0.11) or P3a amplitude (p = 0.12).

In ConstantIgnore, phoneme changes elicited a statistically significant MMN and a P3a in both groups (Table 1). There were no statistically significant group differences in RM-ANOVAs (for MMN, p = 0.58; for P3a, p = 0.13).

### Phoneme perception in variable context

Most participants in both dyslexic and control groups demonstrated explicit awareness of the phoneme change, as they could verbally describe the phoneme change after hearing it in VariableIgnore and then in a short familiarization period of attentive listening (VariableFamiliarization), at least to some extent (Table 2). There were no statistically significant group differences in the scores given to their verbal responses (independent-samples Mann-Whitney U tests after VariableIgnore p = 0.30, after VariableFamiliarization p = 0.20).

**Table 2:** Verbal response scores.

	Group	0 points	1 point	2 points	Total
After VariableIgnore	Dyslexics	6	6	6	18
	Controls	5	4	11	20
After VariableFamiliarization	Dyslexics	3	7	8	18
	Controls	2	4	14	20

*Note*. The table shows number of participants who demonstrated no awareness (0 points), some awareness (1 point), and complete awareness (2 points) of the vowel deviant after VariableIgnore (ignore condition) and VariableFamiliarization (attentive condition). Ten control participants’ data were reported in^44^.

Both groups performed above chance level in behavioral detection of the phoneme changes in VariableAttend (one-sample t-tests reported in Table 3). Dyslexics had statistically significantly lower hit-ratios than controls (independent-samples Mann-Whitney U test, U = 98.50, p = 0.03), whereas no group differences were found for reaction times (p = 0.96).

**Table 3:** Mean hit (H) and false alarm (FA) percentages, hit-ratios (HR), and reaction times (RT) during VariableFamiliarization and VariableAttend.

Sequence	Group	H, %	FA, %	HR, %		RT, ms	
				Mean	Range	Mean	Range
VariableFamiliarization^a^	Dyslexics	77.2 (28.4)	5.2 (5.7)	69.7 (29.6)	0.0–100.0	587.5 (233.7)	308.7–1073.4
	Controls	97.2 (6.1)	4.5 (6.0)	80.1 (22.0)	42.9–100.0	521.3 (118.4)	374.6–791.2
VariableAttend	Dyslexics^b^	86.3 (22.8)	4.4 (8.6)	78.6 (28.8)***	11.7–100.0	460.3 (88.2)	321.0–672.9
	Controls	99.2 (2.0)	2.9 (9.9)	92.9 (20.6)***	21.1–100.0	453.7 (72.4)	342.0–596.0

*Note*. Standard deviation is in parentheses. Hit percentages are calculated as hits per targets and false alarm percentages as false alarms per standards. Hit-ratios in VariableAttend differing statistically significantly from chance-level (10%) are marked with asterisks. Ten control participants’ data were reported in^44^.

^a^In VariableFamiliarization, the instruction was to react to both deviant types, and thus hit-ratio was calculated as hit-% per button presses that were not hits to the other deviant (rule violation). No statistical tests were conducted for this sequence.

^b^One dyslexic participant demonstrated an extremely low hit percentage in VariableAttend despite good performance in VariableFamiliarization. This was interpreted as a misunderstanding of task instructions and the data of that participant in VariableAttend was omitted from analyses.

***p < 0.001, in one-sample t-tests against chance-level (10.0). All tests survive the Bonferroni-corrected critical p-value of 0.01.

## Discussion

The present study demonstrated deficient neural discrimination of phoneme changes in dyslexia in a variable auditory context, evident in the MMN amplitude in the ignore listening condition (VariableIgnore) as well as in the MMN/N2b amplitude in the attentive listening condition (VariableAttend). In addition, there was a trend of a diminished P3a in the dyslexic compared to control group (in the VariableIgnore condition). Dyslexics were also less accurate than controls in behavioral detection of the phoneme changes in the VariableAttend condition. In contrast, no significant group differences were observed in the MMN or P3a amplitudes when the phoneme change was presented in the ConstantIgnore condition without acoustic variation. These findings demonstrate a marked deficit in dyslexics’ ability to neurally and perceptually discriminate between acoustically distinctive native language phonemes in the presence of *f*_o_ variation, consistent with our hypothesis. We propose that these results offer evidence for neural level categorical phoneme processing deficits that may underlie the reading problems in dyslexia.

Acoustic variation is an essential part of natural speech due to, e.g., differences between speakers and prosodic changes. Previous work has shown that it does not compromise neural auditory processing of native language speech sounds in healthy adults and newborns^2,3,44^, interpreted as categorical phoneme processing in adults and a readiness to these functions at birth. However, in light of our results, such variation may pose a challenge to auditory processing in dyslexics, as indicated by their diminished MMN/N2b in the VariableIgnore and VariableAttend but not in the ConstantIgnore condition. Discriminating large acoustic differences in constant contexts is known to be less affected or even normal-like in dyslexia^35^.

Settings with high acoustic variation are more likely to require categorical phoneme processing than ones with repetitive stimulation, and thus, our results could reflect compromised categorical phoneme processing^40–43^, being in line with the allophonic perception theory of dyslexia^43,45,46^. According to the theory, the categorical perception difficulty in dyslexia is associated with a heightened sensitivity to differences between allophones, variants of phonemes that still belong to the same phoneme category in a given language^43,45,46^. The allophonic perception theory of dyslexia (in at-risk children^47^; in dyslexic children^48^) has been tested by recording MMN to a within-category deviant (allophonic contrast: an allophone of the same phoneme), and to a between-category deviant (phonemic contrast: different phoneme). The results demonstrated neural discrimination of within-category deviants only in dyslexics^47^ or enhanced discrimination of between-category deviants in controls^47,48^. In one study, the MMN findings were also paralleled by behavioral results showing that Mandarin-speaking dyslexic children were deficient in an identification task presenting lexical tones as a continuum (exemplars of the same lexical tones were presented in the MMN paradigm^48^).

Based on the allophonic perception theory, the present results may also have important implications for understanding the developmental trajectory of dyslexia. During early language development, certain allophonic features are grouped together to make language-specific neural representations of phoneme categories^1,45,49^. Should this development not succeed, the result could be the core dysfunction in dyslexia: weak formation of accurate phoneme representations^8,11,12^. Future research in infants at familial risk for dyslexia should investigate whether their neural phoneme discrimination ability is compromised in variable acoustic contexts that most likely call for categorical processing. If this deficit is associated with phonological problems later in development, it would support the association suggested above.

Whereas the results of the present experiment have been interpreted to be in line with the allophonic theory of dyslexia, another promising theoretical framework for them stems from the procedural learning hypothesis of dyslexia and related language disorders (e.g.^50^). Gabay *et al*. have demonstrated that dyslexics have difficulties in incidental learning of auditory categories and statistical regularities^51,52^, and suggested that these could indicate broad implicit (or procedural) learning problems that may be the underlying cause for the poor acquisition of native language phoneme categories in dyslexia. Future research will show, whether the dyslexics’ deficits in processing variable auditory information in the present study stem from general challenges in implicit or categorical processing, or from anomalies more specific to speech and language networks of the brain^11^.

When interpreting the constant context results of our study, it is also relevant to note the fixed order of the experimental sequences, with the VariableIgnore and VariableAttend conditions always presented prior to the ConstantIgnore condition. The reason for this choice was the large and salient acoustical difference between the naturally uttered /i/ and /ae/ phonemes in the ConstantIgnore condition. Had it been presented earlier during the experiment, the stimuli would have caught the participants’ attention and could have aided the processing of the phoneme change in the following Variable conditions. Due to the fixed order of sequences, a learning effect in the dyslexic but not the control group (e.g., due to a ceiling effect) during the experiment could explain or contribute to the lack of group difference in the ConstantIgnore condition. This explanation is, however, unlikely for several reasons. Firstly, the phoneme contrast chosen for the study has a large acoustic difference, processing of which should be little affected by dyslexia based on previous work^35^. Secondly, dyslexics have been found to have specific difficulties in neural auditory learning^53–55^. Even so, due to the possibility of, for example, learning or fatigue effects in the ConstantIgnore condition, the neural responses in the Constant and Variable conditions were not directly statistically compared in this study.

Our results showing diminished MMN/N2b responses among dyslexics also during attentive listening are in line with our hypothesis and a previous study showing problems in dyslexics’ neural auditory discrimination also at the attentive processing level, evidenced by an absent N2b response to vowel duration changes in dyslexics^32^. Indeed, by visual inspection of the ERP waveforms in Fig. 2, it seems that the N2b response might be absent in the dyslexic group of the present study. The results of the statistical tests support this by showing a significant group difference at the MMN/N2b latency but not at the earlier MMN latency. It is noteworthy that during the VariableAttend condition, the participants were instructed to detect and respond to another deviant type, namely, violations of a complex rule in the auditory stream. A demanding primary task may diminish discriminative responses to the unattended deviants (e.g., P3a^56^), possibly particularly in the dyslexic group if the task was more demanding for them. This could even explain the lack of an MMN enhancement or an N2b in the dyslexic group in the VariableAttend condition. However, our finding on impaired perceptual discrimination of the phoneme changes is compatible with the interpretation of a genuine deficit in attentive processing of the phoneme changes in a variable context.

The current study also found an insignificant trend for the P3a amplitude to be diminished in dyslexics in the VariableIgnore condition, consistent with a previous study^33^. This reduced P3a amplitude, indicating poor attention shifting to changes, might result from insufficient change discrimination as suggested by the diminished MMN in these participants. While this insignificant effect had a reasonable effect size, the robustness of this finding should be confirmed by future research.

As expected, both dyslexics and controls could verbally describe the phoneme change in the acoustically variable context when queried about them after stimulus presentation, with no statistically significant differences in response scores between the groups. The result is not surprising, as a failure to detect them would indicate severe hearing- or language-related difficulties, not reported by the dyslexics of the present study. Similarly, behavioral detection of the phoneme changes in the variable context as indicated by hit-ratios during VariableAttend condition was clearly above chance level in both groups. However, dyslexics were significantly less accurate than controls in phoneme change detection, which emerged as a lower hit rate but not in the reaction time. Thus, the compromised processing of native language phonemes in a variable context in dyslexia was evident even at the perceptual level, which is rather striking when taking into account the large acoustic difference between the two vowels. Perhaps dyslexics’ difficulties in correctly reacting to deviant phonemes were due to the requirement to rapidly press a button to them when they occurred among stimuli presented with a rather fast pace, which is challenging to their auditory system^37,57^.

The present results highlight the huge challenge of normal speech listening conditions for the speech system of dyslexic individuals: in a variable auditory context mimicking natural variability of speech, dyslexics detect poorly even the most salient phoneme differences. This may even stem from an innate difficulty in perceiving or implicitly adopting auditory categories, either in speech or more generally in complex (auditory) material. This difficulty could compromise the acquisition of native language phoneme representations during language learning in infancy, and thus underlie the phonological deficits that are considered the core difficulties in dyslexia^8,11,12^. The results also suggest that simple experimental paradigms with repetitive stimuli can be insensitive to the difficulties underlying developmental dyslexia. This should be taken into account in future neurophysiological studies aiming to find out which processes are intact and which ones are deficient in dyslexics. This is highly relevant in order to find the optimally sensitive markers of language and reading disorders in different age and severity groups, which is a major challenge in the field.

## Methods

### Participants

The study included altogether 39 participants; one participant was excluded due to having absolute pitch. The participants in the dyslexic group (n = 18, nine males, mean age 32.5 years, SD 8.9 years) had to have their reading speed or accuracy below one SD from the population mean in at least two out of three reading tests: word list reading, pseudoword list reading, and reading aloud a narrative text^58^ (standardized based on a control data^59^). Most participants (17 out of 18) were least 2 SD below the population mean in at least one reading test. Second, their symptoms had to date back to childhood (based on an interview or the Adult Reading History Questionnaire, ARHQ^60^). The control group participants reported no history of reading difficulties (n = 20, 10 males, mean age 29.5 years, SD 7.9 years). All participants reported being right handed, native monolingual Finnish speakers with normal hearing, normal or corrected-to-normal vision, without other neurological or psychiatric conditions than dyslexia in the dyslexic group. Attention-related impairments were screened for with Adult ADHD Self-Report Scale Symptom Checklist (ASRS-v1.1^61^). The control and dyslexic groups did not differ statistically significantly in age, gender distribution, or education level (chi square test for gender p > 0.99, and for education level p = 0.47; independent samples t-test for age p = 0.28).

The participants gave a written informed consent and received a compensation for their participation (vouchers for cultural or exercise activities). Ethical approval was received from the University of Helsinki Review Board in Humanities and Social and Behavioural Sciences, and the study was conducted in accordance with the declaration of Helsinki. Part of the data of 10 control participants (approximately 26% overlap in the participant sample) were also reported in a previous study^44^.

### Experimental stimuli and paradigms

**Figure 4:**
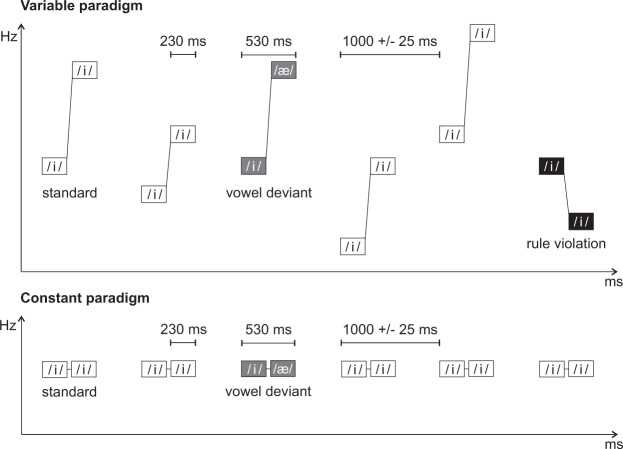
Experimental paradigms. Adapted from Virtala *et al*.^44^.

The stimuli were Finnish phonemes /i/ and /æ/ uttered by a female Finnish native speaker, chosen based on their large acoustic difference^62^ and edited with Praat 5.4.01^63^ and Adobe Audition CS6 5.0. Build 708 (Adobe Systems Inc., California, USA). Sound intensity levels were root-mean-square (RMS) normalized between the phonemes, and the phonemes were cut from the end with natural attack retained and the ending modified at 190–230 ms with smooth fade out function in Adobe Audition, resulting in a 230-ms duration. The phonemes were transposed to seven frequency levels one to three semitones lower (174.3, 184.6, and 195.4 Hz) and one to four semitones higher (217.8, 229.7, 242.5, and 256.2 Hz) than the natural *f*_o_-level of 206.8 Hz. Each phoneme thus had eight *f*_o_ frequency levels in a typical range for female speech. The phonemes were presented as stimulus pairs /i/-/i/ or /i/-/æ/ (530-ms duration and a 70-ms silent gap in between) in a variable and a constant oddball paradigm (Fig. 4). The variable paradigm was created to probe two distinct neural processes: phoneme discrimination, on the one hand, and detection of rule-based auditory regularities, on the other (introduced in Virtala *et al*.^44^). The data concerning rule detection (/i/-/i/ pairs) will be reported elsewhere.

The variable paradigm included 42 /i/-/i/ pairs (21 with rising, 21 with falling frequency) and 21 /i/-/æ/ pairs (with rising frequency) with all possible frequency combinations except frequency difference within a pair being always > 1 semitone. The /i/-/i/ pairs with a rising frequency served as the standards (probability 80%), while /i/-/æ/ pairs with a rising frequency (vowel deviants, 10%), and /i/-/i/ pairs with a falling frequency (rule violations, 10%) served as deviants.

The constant paradigm only included phonemes at the natural *f*_o_-level (206.8 Hz). The standard was /i/-/i/ (probability 90%), and the deviant was /i/-/æ/ (vowel deviant, 10%). In both variable and constant paradigms, the stimuli were presented with a 25-ms jitter in 10-ms steps (975, 985… 1025 ms between stimulus onsets in order to reduce phase-locked neural activity to the predictable stimulus onsets) pseudo-randomly, with at least one standard preceding every deviant.

### Experimental procedure

EEG was recorded during stimulus presentation via headphones (Sony Dynamic Stereo Headphones, MDR-7506), in two sessions, while the participant sat in a soundproof, electrically shielded chamber. Stimuli were presented at approximately 65 dB SPL(a) (Presentation software, NeuroBehavioral Systems Inc., California, USA), version 17.2, and behavioral responses were recorded with Cedrus RB844 response pad (Cedrus Corporation, California, USA). In ignore conditions the participant watched a subtitled soundless movie, and was instructed not to pay attention to the sounds. In attentive conditions the movie was turned off, and the participant was instructed to pay attention to the sounds, and press a response button to target deviants.

An overview of the experimental protocol can be found in Table 4. First, the participants heard the variable paradigm in a 21-minute long sequence in an ignore condition (VariableIgnore, 1260 stimuli, 126 vowel deviants), and then in a 90-second long attentive familiarization session (VariableFamiliarization, 90 stimuli, 9 vowel deviants). Before and after the session, participants were queried about the nature of the two deviants in order to orientate the participants to the detection task and to study whether explicit awareness of the deviants arises. Then they were informed about the nature of the deviants, and had to detect first one and then the other of them while the variable paradigm was presented in two ~10-min sequences in counter-balanced order in an attentive condition (VariableAttend, two blocks, each with 630 stimuli, 63 vowel deviants). Finally, the constant paradigm was presented in a 21-min-long sequence in ignore condition (ConstantIgnore, 1260 stimuli, 126 deviants). The first two (VariableIgnore, VariableFamiliarization) and the last sequence (ConstantIgnore) were always presented in the same order, in order to minimize the participants’ explicit awareness of the nature of the stimuli during the presentation of the variable paradigms.

**Table 4:** Experimental protocol.

Condition	EEG	Given instructions (I), correct answers (A), and queries (Q)
VariableIgnore	Yes	I: *“Your task is to focus on the silent movie and ignore the presented sound stream.”*
		Q: *“The sound stream you heard varies constantly, but occasionally, two types of changes occur in it. Can you name these changes in the sound stream?”*
VariableFamiliarization	No	I: *“Next, focus on listening to the sound stream and think what changes occur in it. Push the response box button always immediately after hearing a change.”*
		Q: *“Can you now tell, what kind of changes occur in the sound stream?”*
		A: *“In the stream of speech sounds, there are vowel changes (from /i/ to /* æ/*) and rule violations as follows: when in most of the vowel pairs, pitch rises so that first sound is lower than the next sound, in some vowel pairs it falls, so that first sound is higher than the next sound.”*
VariableAttend	Yes	I: *“Again, focus on listening to the sound stream. Push the response box button always immediately after hearing the vowel change. Ignore the rule violation.”*
VariableAttend	Yes	I: *“Again, focus on listening to the sound stream. Push the response box button always immediately after hearing the rule violation. Ignore the vowel change.”*
ConstantIgnore	Yes	I: *“Your task is to focus on the silent movie and ignore the presented sound stream.”*

EEG was recorded during all sequences except VariableFamiliarization, and the participant was instructed to avoid moving or blinking and to minimize eye movements by, for example, fixating eyes on a self-chosen point at the screen in front of him/her.

### EEG recording and analysis

The EEG (sampling rate 512 Hz) was recorded using the BioSemi system (headcap and amplifier: Biosemi ActiveTwo mk2, BioSemi B. V., Amsterdam, The Netherlands) with a 64-active-electrode cap, referenced to the CMS, and electrodes placed according to the international 10–20-system. External Ag/AgCl electrodes were placed on left and right mastoids and nose, as well as below and at the outer corner of the right eye to record the vertical (VEOG) and horizontal electro-oculogram (HEOG), respectively.

Data analyses were performed with BESA Research 6.0 Software (BESA GmbH, Gräfelfing, Germany). Data were offline re-referenced to the average of mastoid electrodes and filtered at 1–30 Hz (slope 12 dB/oct., zero phase). Peripheral electrodes (close to the edge of the electrode cap) including a lot of high-frequency noise were excluded from the data and other noisy electrodes replaced with the interpolating algorithm in BESA 6.0 (max. three interpolated electrodes per participant per condition).

The EEG was epoched into −100–975-ms time windows at phoneme pair onset with a −100–0-ms baseline correction. An automatic eye-blink correction was performed (detection thresholds 150 μV for horizontal, 250 μV for vertical eye movements, principal component analysis components corresponding to eye movements were subtracted from the original EEG signal, BESA v 6.0^64^). Epochs with other high-amplitude artifacts exceeding ±120 μV were automatically removed. Epochs were averaged separately for each sequence, participant, stimulus type, and electrode. Difference waves were calculated by subtracting the response elicited by the standards from those elicited by the deviants.

All participants had at least 80% accepted epochs from each sequence. The average amounts of deviant trials per sequence were: in VariableIgnore, dyslexics 123 (range 110–126) and controls 125 (range 120–126); in VariableAttend, 62 (56–63) and 63 (61–63); in ConstantIgnore, 124 (121–126) and 125 (124–126). The responses to vowel deviants were analyzed in that block of the VariableAttend sequence where the vowel deviants did not serve as targets, responses in the other block most likely including confounding motor artefacts in the EEG signal due to button presses.

### Quantification and statistical analysis of EEG and behavioral data

Latencies of MMN/N2b (N2b contribution expected in the attentive conditions) and P3a peak amplitudes were assessed from individual participants from the midline electrodes (Fz, FCz, and Cz) using the time windows (measured from the onset of the second phoneme, the deviance onset) of 100–300 ms for MMN/N2b and 150–450 ms for P3a, based on previous literature and visual inspection of the ERP waveforms. RM-ANOVA was separately conducted for MMN/N2b and P3a latencies: Group (dyslexia/control) x Electrode (Fz, FCz, Cz).

No group differences were found for the latencies (in all p > 0.05), and thus the same 50-ms time windows centered at the group mean peak latencies were used for both groups to calculate mean amplitudes for MMN/N2b and P3a. In VariableAttend, the MMN was calculated from the same time window as in VariableIgnore, as the MMN in the attentive condition was considered to overlap with other, attention-related components (mainly N2b), and an additional time window was centered at the actual peak of the response (termed MMN/N2b). One-sample two-tailed t-tests with Bonferroni-corrections were used to determine the presence of MMN, MMN/N2b, and P3a at Fz electrode (two components, two groups, and three sequences, plus MMN/N2b in VariableAttend, 14 t-tests in total). Group effects on MMN, MMN/N2b, and P3a amplitudes were analyzed in RM-ANOVAs with group as a between-subject’s factor (two components, three sequences, plus MMN/N2b, seven RM-ANOVAs in total). Nine electrodes close to the fronto-central midline (F1, Fz, F2, FC1, FCz, FC2, C1, Cz, C2) were included in all the RM-ANOVAs (within-subjects factor: electrode) in order to improve the signal-to-noise-ratio; however, the spatial distribution of the components was not analyzed. When sphericity assumption was violated, Greenhouse-Geisser correction was used. Effect sizes are reported using partial eta squared (ηp²). Four participants in control group and one in dyslexic group had no data from ConstantIgnore sequence that was added to the paradigm later on.

Verbal responses after VariableIgnore and VariableFamiliarization were scored as 0 (no answer or incorrect answer), 1 (partially correct answer, such as ‘vowels changed’), or 2 (correct answer, e.g., ‘vowel changed from /i/ to /æ/’) and compared between groups with nonparametric independent samples Mann-Whitney U test due to their skewed distributions. Behavioral performance in VariableFamiliarization and VariableAttend was quantified as hit-ratios (hit-% per button presses) and reaction times (in ms). In VariableFamiliarization, the instruction was to react to both deviant types, and thus hit-ratio was calculated as hit-% per button presses that were not hits to the other deviant (rule violation). Because this sequence was also very short, hit-ratio and reaction time were not statistically analyzed. Hit-ratio in VariableAttend was compared against chance-level (10%, probability of the vowel deviant in the sequence) with a one-sample t-test. Hit-ratio and reaction time in VariableAttend were compared between groups with non-parametric independent samples Mann-Whitney U tests, as they were not normally distributed [hit-rate, Shapiro-Wilk(37) = 0.63, p < 0.001; reaction time, Shapiro-Wilk(37) = 0.95, p = 0.11].

## Data Availability

As no common repositories for neurocognitive data are available for the authors and the ethical permission does not include a clause determining the specifics of data availability, the data can be provided for readers upon reasonable request to the first author, as is common practice in the field.

## References

[1] Kuhl PK (2004). Early language acquisition: cracking the speech code. Nat. Rev. Neurosci..

[2] Shestakova A (2002). Abstract phoneme representations in the left temporal cortex: magnetic mismatch negativity study. Neuroreport.

[3] Dehaene-Lambertz G, Pena M (2001). Electrophysiological evidence for automatic phonetic processing in neonates. Neuroreport.

[4] Kere J (2014). The molecular genetics and neurobiology of developmental dyslexia as model of a complex phenotype. Biochem. Biophys. Res. Commun..

[5] Peterson RL, Pennington BF (2015). Developmental dyslexia. Annu. Rev. of Clinical Psychol..

[6] Lyon GR, Shaywitz SE, Shaywitz BA (2003). A Definition of Dyslexia. Ann. Dyslexia.

[7] Shaywitz SE (1998). Dyslexia. N. Engl. J. Med..

[8] Eden Guinevere F., Olulade Olumide A., Evans Tanya M., Krafnick Anthony J., Alkire Diana R. (2016). Developmental Dyslexia. Neurobiology of Language.

[9] Galaburda AM, LoTurco J, Ramus F, Fitch RH, Rosen GD (2006). From genes to behavior in developmental dyslexia. Nat. Neurosci..

[10] Kere J (2011). Molecular genetics and molecular biology of dyslexia. Wiley Interdiscip. Rev. Cogn. Sci..

[11] Giraud AL, Ramus F (2013). Neurogenetics and auditory processing in developmental dyslexia. Curr. Opin. Neurobiol..

[12] Vellutino FR, Fletcher JM, Snowling MJ, Scanlon DM (2004). Specific reading disability (dyslexia): what have we learned in the past four decades?. J. Child Psychol. Psychiatry.

[13] Boets B (2013). Intact but less accessible phonetic representations in adults with dyslexia. Science (80-)..

[14] Hämäläinen JA, Salminen HK, Leppänen PHT (2013). Basic Auditory Processing Deficits in Dyslexia: Systematic Review of the Behavioral and Event-Related Potential/ Field Evidence. J. Learn. Disabil..

[15] Kujala T, Näätänen R (2001). The mismatch negativity in evaluating central auditory dysfunction in dyslexia. Neurosci. Biobehav. Rev..

[16] Kujala TM (2007). The role of early auditory discrimination deficits in language disorders. J. Psychophysiol..

[17] Kujala T, Tervaniemi M, Schröger E (2007). The mismatch negativity in cognitive and clinical neuroscience: Theoretical and methodological considerations. Biol. Psychol..

[18] Näätänen R, Kujala T, Winkler I (2011). Auditory processing that leads to conscious perception: A unique window to central auditory processing opened by the mismatch negativity and related responses. Psychophysiology.

[19] Näätänen R, Simpson M, Loveless NE (1982). Stimulus deviance and evoked potentials. Biol. Psychol..

[20] Alho K (1998). Processing of novel sounds and frequency changes in the human auditory cortex: Magnetoencephalographic recordings. Psychophysiology.

[21] Horváth J, Winkler I, Bendixen A (2008). Do N1/MMN, P3a, and RON form a strongly coupled chain reflecting the three stages of auditory distraction?. Biol. Psychol..

[22] Näätänen R (1997). Language-specific phoneme representations revealed by electric and magnetic brain responses. Nature.

[23] Näätänen R (2001). The perception of speech sounds by the human brain as reflected by the mismatch negativity (MMN) and its magnetic equivalent (MMNm). Psychophysiology.

[24] Kujala T, Leminen M (2017). Low-level neural auditory discrimination dysfunctions in specific language impairment—A review on mismatch negativity findings. Dev. Cogn. Neurosci..

[25] Maurer U (2009). Neurophysiology in preschool improves behavioral prediction of reading ability throughout primary school. Biol. Psychiatry.

[26] Van Zuijen TL (2012). Temporal auditory processing at 17 months of age is associated with preliterate language comprehension and later word reading fluency: An ERP study. Neurosci. Lett..

[27] Schaadt G, Männel C (2019). Phonemes, words, and phrases: Tracking phonological processing in pre-schoolers developing dyslexia. Clin. Neurophysiol..

[28] Bitz U, Gust K, Spitzer M, Kiefer M (2007). Phonological deficit in school children is reflected in the mismatch negativity. Neuroreport.

[29] Lachmann T, Berti S, Kujala T, Schröger E (2005). Diagnostic subgroups of developmental dyslexia have different deficits in neural processing of tones and phonemes. Int. J. Psychophysiol..

[30] Lovio R, Näätänen R, Kujala T (2010). Abnormal pattern of cortical speech feature discrimination in 6-year-old children at risk for dyslexia. Brain Res..

[31] Schulte-Körne G, Deimel W, Bartling J, Remschmidt H (2001). Speech perception deficit in dyslexic adults as measured by mismatch negativity (MMN). Int. J. Psychophysiol..

[32] Kujala T (2006). Speech- and sound-segmentation in dyslexia: Evidence for a multiple-level cortical impairment. Eur. J. Neurosci..

[33] Fosker T, Thierry G (2004). P300 investigation of phoneme change detection in dyslexic adults. Neurosci. Lett..

[34] Corbera S, Escera C, Artigas J (2006). Impaired duration mismatch negativity in developmental dyslexia. Neuroreport.

[35] Baldeweg T, Richardon A, Watkins S, Foale C, Gruzelier G (1999). Impaired auditory frequency discrimination in dyslexia detected with mismatch evoked potentials. Ann. Neurol..

[36] Bishop DVM (2007). Using mismatch negativity to study central auditory processing in developmental language and literacy impairments: Where are we, and where should we be going?. Psychol. Bull..

[37] Kujala T (2000). Basic auditory dysfunction in dyslexia as demonstrated by brain activity measurements. Psychophysiology.

[38] Kujala T, Belitz S, Tervaniemi M, Näätänen R (2003). Auditory sensory memory disorder in dyslexic adults as indexed by the mismatch negativity. Eur. J. Neurosci..

[39] Kujala T, Lovio R, Lepistö T, Laasonen M, Näätänen R (2006). Evaluation of multi-attribute auditory discrimination in dyslexia with the mismatch negativity. Clin. Neurophysiol..

[40] Werker JF, Tees RC (1987). Speech perception in severely disabled and average reading children. Can. J. Psychol..

[41] Bogliotti C, Serniclaes W, Messaoud-Galusi S, Sprenger-Charolles L (2008). Discrimination of speech sounds by children with dyslexia: Comparisons with chronological age and reading level controls. J. Exp. Child Psychol..

[42] Godfrey JJ, Syrdal-Lasky K, Millay KK, Knox CM (1981). Performance of dyslexic children on speech perception tests. J. Exp. Child Psychol..

[43] Noordenbos MW, Serniclaes W (2015). The categorical perception deficit in dyslexia: A meta-analysis. Sci. Stud. Read..

[44] Virtala P, Partanen E, Tervaniemi M, Kujala T (2018). Neural discrimination of speech sound changes in a variable context occurs irrespective of attention and explicit awareness. Biol. Psychol..

[45] Serniclaes W (2018). Allophonic theory of dyslexia: A short overview. JSM Commun. Disord..

[46] Serniclaes, W. & Sprenger-Charolles, L. Categorical perception of speech sounds and dyslexia. *Curr. Psychol. Lett. Behav. brain Cogn*. **1** (2003).

[47] Noordenbos MW, Segers E, Serniclaes W, Mitterer H, Verhoeven L (2012). Neural evidence of allophonic perception in children at risk for dyslexia. Neuropsychologia.

[48] Zhang Y (2012). Universality of categorical perception deficit in developmental dyslexia: An investigation of Mandarin Chinese tones. J. Child Psychol. Psychiatry Allied Discip..

[49] Hoonhorst I (2009). French native speakers in the making: From language-general to language-specific voicing boundaries. J. Exp. Child Psychol..

[50] Lum JAG, Ullman MT, Conti-Ramsden G (2013). Procedural learning is impaired in dyslexia: Evidence from a meta-analysis of serial reaction time studies. Res. Dev. Disabil..

[51] Gabay Y, Holt LL (2015). Incidental learning of sound categories is impaired in developmental dyslexia. Cortex.

[52] Gabay Y, Thiessen ED, Holt LL (2015). Impaired statistical learning in developmental dyslexia. J. Speech, Lang. Hear. Res..

[53] Kimppa L, Shtyrov Y, Partanen E, Kujala T (2018). Impaired neural mechanism for online novel word acquisition in dyslexic children. Sci. Rep..

[54] Perrachione TK (2016). Dysfunction of rapid neural adaptation in dyslexia. Neuron.

[55] Ahissar M (2007). Dyslexia and the anchoring-deficit hypothesis. Trends Cogn. Sci..

[56] Harmony T (2000). Primary task demands modulate P3a amplitude. Cogn. Brain Res..

[57] Nagarajan S (1999). Cortical auditory signal processing in poor readers. Proc. Natl. Acad. Sci..

[58] Nevala, J., Kairaluoma, L., Ahonen, T., Aro, M. & Holopainen, L. Lukemis- ja kirjoittamistaitojen yksilötestistö nuorille ja aikuisille [Individual test material for assessing dyslexia in youth and in adult age] (Standardization version). (2006).

[59] Laasonen M, Lehtinen M, Leppämäki S, Tani P, Hokkanen L (2010). Project DyAdd: Phonological processing, reading, spelling, and arithmetic in adults with dyslexia or ADHD. J. Learn. Disabil..

[60] Lefly DL, Pennington BF (2000). Reliability and validity of the adult reading history questionnaire. J. Learn. Disabil..

[61] Kessler RC (2005). The World Health Organization Adult ADHD Self-Report Scale (ASRS): a short screening scale for use in the general population. Psychol. Med..

[62] Wiik, K. *Finnish and English vowels. A comparison with special reference to the learning problems met by native speakers of Finnish learning English*. (Publications of University of Turku, 1965).

[63] Boersma, P. & Weenink, D. Praat: Doing phonetics by computer. Retrieved from http://www.praat.org/ (2013).

[64] Ille N, Berg P, Scherg M (2002). Artifact correction of the ongoing EEG using spatial filters based on artifact and brain signal topographies. J. Clin. Neurophysiol..

